# CHoosing Active Role Models to INspire Girls (CHARMING): protocol for a cluster randomised feasibility trial of a school-based, community-linked programme to increase physical activity levels in 9–10-year-old girls

**DOI:** 10.1186/s40814-021-00961-6

**Published:** 2022-01-03

**Authors:** Bethan Pell, Jemma Hawkins, Rebecca Cannings-John, Joanna M. Charles, Britt Hallingberg, Graham Moore, Joan Roberts, Esther van Sluijs, Kelly Morgan

**Affiliations:** 1 Centre for Development, Evaluation, Complexity and Implementation in Public Health Improvement (DECIPHer), Cardiff University, Cardiff, UK; 2 Centre for Trials Research, Cardiff University, Heath Park, CF14 4YS UK; 3 Centre for Health Economics and Medicines Evaluation, Bangor University, Normal Site, Holyhead Road, Bangor, Gwynedd LL57 2PZ UK; 4 Cardiff School of Sport and Health Sciences, Cardiff Metropolitan University, Cardiff, UK; 5 MRC Epidemiology Unit & Centre for Diet and Activity Research, University of Cambridge School of Clinical Medicine, Box 285, Institute of Metabolic Science, Cambridge Biomedical Campus, Cambridge, CB2 0QQ UK

**Keywords:** Physical activity, Children, Primary school

## Abstract

**Background:**

In the UK, there is evidence that girls’ physical activity tends to decline to a greater extent than boys as they enter adolescence. ‘Role models’ could play a vital role in inspiring girls to become or remain physically active. The CHARMING Programme is a primary school-based community linked role-model programme, co-developed in 2016, with children, parents, schools and wider stakeholders. It involves different types of physical activity delivered for 1-h each week by a community provider and peer role models (e.g. older girls from secondary schools) joining in with the sessions. The programme ultimately aims to increase and sustain physical activity levels among 9–10-year-old girls. This study aims to assess the feasibility and acceptability of the CHARMING Programme and of evaluating it using a randomised trial.

**Methods:**

This study is a feasibility cluster randomised controlled trial, with embedded process evaluation and health economic evaluation. Approximately 90 Year 5 (i.e. 9–10-year-old) girls will be recruited across six primary schools in Mid-South Wales. Participating schools will be allocated to the programme: control on a 2:1 basis; four intervention schools will run the CHARMING Programme and two will continue with usual practice. A survey and accelerometer will be administered at baseline and repeated at 12 months. Interviews and focus groups will be conducted post-intervention delivery. The primary aim is to assess feasibility of a future randomised trial via the recruitment of schools, participants and role models; randomisation; retention; reach; data collection completion rates; programme adherence; and programme fidelity, views on intervention acceptability and programme barriers and facilitators. Secondary aims are to evaluate established physical activity outcome measures for children plus additional health economic outcomes for inclusion in a future full-scale trial.

**Discussion:**

The results of this study will inform decisions on whether and how to proceed to a full-scale evaluation of the effectiveness and cost-effectiveness of the CHARMING Programme to improve or sustain physical activity.

**Trial registration:**

ClinicalTrials.gov ISRCTN36223327. Registered March 29, 2021

**Supplementary Information:**

The online version contains supplementary material available at 10.1186/s40814-021-00961-6.

## Background

Physical activity is important for young people’s health and wellbeing, but most young people are not active enough to encounter health benefits [1]. Young people in Wales are among the least active worldwide, with fewer than 20% of 11–16-year-olds meeting current guidelines of being active 60 min a day [2]. Studies show that girls tend to become less active than boys by age 10 and that this trend continues into secondary school [3]. There is a need for earlier intervention at primary school age, in the transition to adolescence, to prevent population level declines in girls’ physical activity through engaging less active girls in physical activity and maintaining activity levels among those who are already active [4].

Both social and physical environmental factors play a key role in increasing physical activity levels and understanding physical activity choices at the individual and contextual levels is a crucial step to informing the design of interventions aiming to increase physical activity [5]. As such, interventions grounded in a theoretical framework designed to influence such factors and choices may be more effective than those with no specified theory [6]. With differing mechanisms underlying physical activity levels and extracurricular physical activity playing a central part, there is a need to devise different types of intervention approaches for boys and girls aged 8–12 years [7]. Role models are a potential strategy to inspire young people to become involved, or maintain involvement, in physical activity and sport [8]. Adolescent girls were recently shown as more likely to be active if they had a role model who played sport compared to girls with role models who did not play sport [9]. Role models might positively influence behaviour by contributing, along with other factors, to the perception of specific behaviours (e.g. physical activity and sport participation) as attractive, attainable and rewarding experiences [10]. No one individual role model will be suitable for all young girls, but rather choices are made on the basis of exposure to family, peers and sports celebrities [8]. Such an intervention strategy has been internationally endorsed by the World Health Organization (WHO) with specific recommendations for the use of local community role models to increase physical activity among females [11]. The National Institute for Health and Care Excellence (NICE) also recommends that any practitioner leading physical activity initiatives, including teachers and volunteers, should provide appropriate role models [12]. Despite limited research and a lack of robust trials, policy recommendations strongly support the use of role models for tackling rising inactivity levels among girls [12, 13].

### Rationale and previous work

A formative study led by the Principal Investigator [14] adopted a participative community approach with key stakeholders to design and pilot a school-based role model intervention over a 12-month period. Framed by psychological and sociological theory, the intervention, CHARMING (CHoosing Active Role Models to INspire Girls), aims to promote key self-determination theory constructs and social ecological model constructs.

Main findings from this formative evaluation identified that (a) 28% of girls were unable to identify a role model for physical activity prior to the study pilot; (b) schools were a suitable location for the delivery of a community-linked role model programme; (c) role models and desired activities could be sourced from local communities; (d) intervention uptake and attendance was positive (46 out of 64 girls attended; of which 76% attended 5/6 sessions and 39% reported a black and minority ethnic background providing an early indicator that the intervention is unlikely to widen inequalities); (e) teachers perceived the intervention to be beneficial, highlighting increased opportunities for girls to be active, challenging gender norms in school and developing long-term community partnerships; and (f) recommended intervention modifications included; role model warm-up activities, a longer intervention delivery and the provision of consistent role models across sessions.

These results informed decisions on intervention refinement i.e. removing the warm-up activities, increasing the length of the intervention and adding a peer role model component. A lack of positive role models has previously been identified as a barrier to sport participation for girls [15] and data show that young girls in Wales are insufficiently active both on a national and global scale. Our earlier work [14] contributes to our understanding of the profile of physical activity role models of preadolescent girls (highlighting the importance of gender) and suggests potential for delivering a community-linked role-model programme in schools. Still, further work is required to understand the feasibility and acceptability of this on a larger scale with a demographically diverse population. Regarding the logistical considerations of sourcing peer role models, data suggests a greater influence when role models are relatable to the target audience [16]. Hence, prior to a trial of effectiveness, which may be undermined by difficulties sourcing role models or facilitating their attendance in the programme, or wider implementation issues [17], further feasibility testing is required to investigate these issues. In order to do this, and pilot the methods for a full-scale trial, this study will assess feasibility and acceptability of the CHARMING programme and its evaluation design.

The aim of the feasibility RCT is to assess the feasibility and acceptability of the CHARMING programme and proposed trial methodology. The study will aim to contribute to the evidence base on improving physical activity outcomes for 9–10-year-old girls. Importantly, the study will inform decisions on whether and how to proceed to a full-scale evaluation of the effectiveness and cost-effectiveness of CHARMING.

### Study objectives

The objectives of this study are as follows:

1. Identify effective means of recruiting schools, participants and community and peer role models
2. Assess the feasibility of conducting an effectiveness trial, economic evaluation and assess the implementation potential of the intervention
3. Explore the acceptability of the intervention and the influence of school context on intervention implementation
4. Assess the extent to which each of 6 progression criteria for conducting a full-scale trial are met

## Methods

### Trial design

This trial is a cluster randomised-controlled feasibility study, with allocation at the primary school–level and with an embedded process evaluation and health economic evaluation. Primary schools will be recruited and either randomised to run the intervention or continue with their usual practice.

This protocol was developed in line with the Standard Protocol Items: Recommendations for Interventional Trials (SPIRIT) guidelines; the SPIRIT checklist (Additional File 1) and the study schedule displayed in Table 1.

**Table 1:**
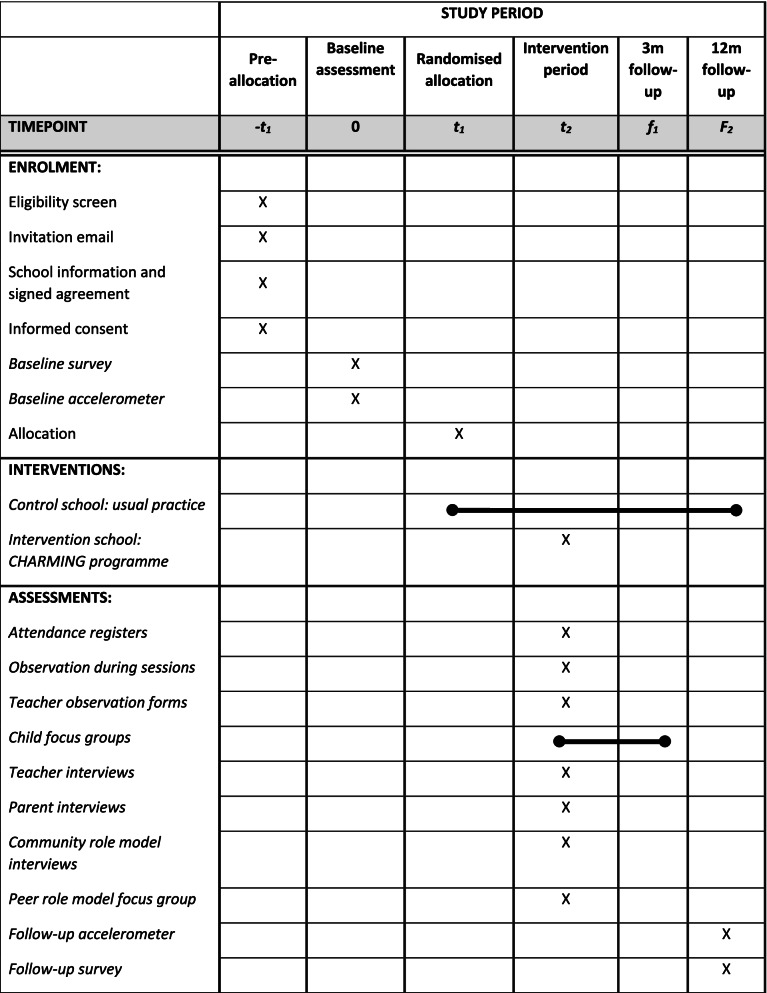
SPIRIT protocol schedule for study timeline

### Study setting

**Fig. 1:**
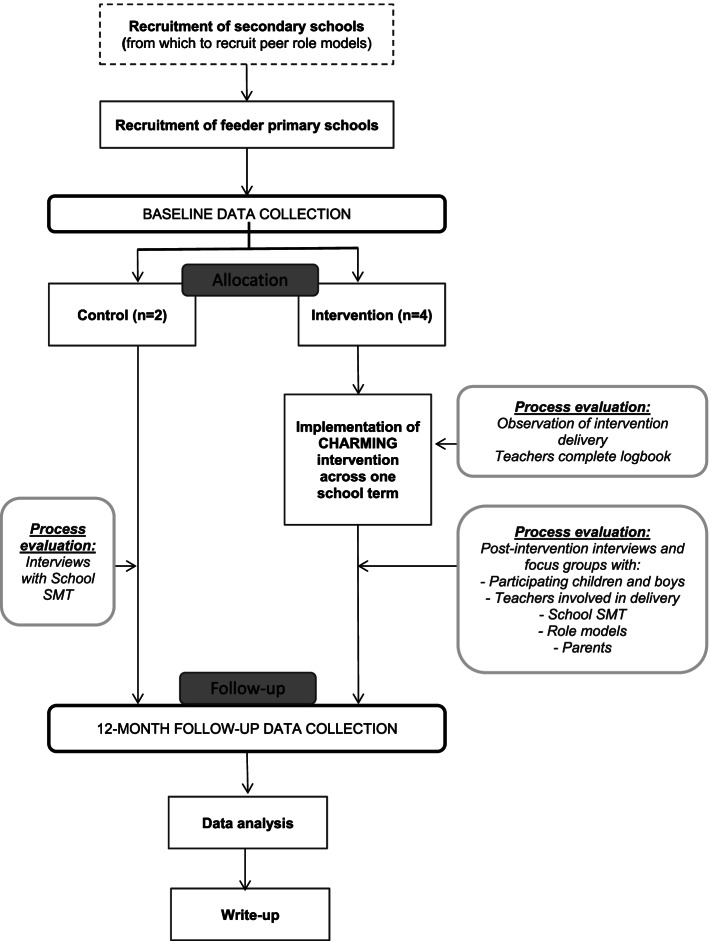
Study flowchart

Six secondary schools across Mid- and South Wales will be recruited into the study, with their adjoining primary schools invited to take part. The study flowchart can be seen in Fig. 1.

### Participants

Participants will be all year 5 girls (aged 9–10) who are opted-in by a parent (with written informed consent) and who provide their own assent.

### Eligibility criteria

Inclusion: All year 5 girls (aged 9–10) who are opted-in by a parent and who provide their own assent.

Exclusion: Children who cannot engage in physical activity due to medical reasons.

### Intervention—CHARMING Programme

The intervention is described using the TIDiER checklist (in Table 2). The intervention is a primary school-based physical activity programme, which consists of five planned components informed by psychological and sociological theory, integrating self-determination theory (SDT) [18] and the socio-ecological model [19]. As such the intervention programme focuses on (i) promoting children’s perceptions of autonomy, relatedness and competence in relation to physical activity and (ii) intrapersonal and interpersonal change within the school organisation and connections to the wider community and community opportunities for physical activity participation. The intervention has been designed to support physical activity engagement via three key psychosocial mechanisms: (1) giving girls the choice of which activities are delivered will support a sense of autonomy, (2) providing both community and peer-role models will increase the likelihood of relatedness and (3) providing a wide-range of activities will support perceived competence within a safe space among female peers. Along with enhanced enjoyment of physical activity, it is anticipated that these mechanisms will improve autonomous motivation, leading to greater maintenance of physical activity. Previous research has shown associations between psychological need satisfaction and autonomous motivation amongst the UK primary school age children [20].

**Table 2:** Intervention description using the Template for Intervention Description and Replication (TIDieR) checklist

1. Brief name	CHoosing Active Role Models to INspire Girls (CHARMING)
2. Why	Less than a quarter of 11–16-year-olds meet current guidelines of being active 60 min a day. Girls are less active than boys and their physical activity levels drop quicker than male peers from the start of adolescence. The transition to adolescence is a crucial time to introduce approaches to support girls to become more active, and help stop the drop in physical activity among those who already are. The use of role models to promote physical activity has been recommended in international guidelines. Most similar studies have focused on older ages and are US-based. Better results have been shown among approaches that target girls only, are based in schools, include a range of activities, use theory and work with girls to produce a programme. A primary school-based role model programme, CHARMING, has been designed to increase and sustain physical activity levels among 9–11-year-old girls. The intervention is underpinned by psychological and sociological theory, integrating self-determination theory and the socio-ecological model.
3. What materials	Participants will have access to materials provided by the community role models following each session. Materials will signpost girls and parents to opportunities to continue the activity in their community using leaflets and usual school communication channels.
4. What procedures	Access to the intervention will be provided to schools upon randomisation to the intervention group. Schools receiving the intervention will run a 6-week programme consisting of five core planned components: 1-h taster physical activity sessions (1 per week after-school on school premises); community role model delivering the session, peer-role model(s) participating in each session, question and answer opportunity with role models post-session and sign-posting to community physical activity opportunities. All schools will have email access to the trial manager for any further enquiries. The linked secondary school recruits a group of secondary school students to act as ‘peer role models’ for the intervention [i.e. a females aged 11–16 years who young girls might look up to as a role model for physical activity] to participate in the weekly sessions in the primary school and support girls’ engagement with the intervention.
5. Who provided	The study manager will provide community role models and peer role models with a roles and responsibilities document. A recruitment poster will also be provided to the secondary school as a means of advertising the opportunity to be involved in the programme. The teacher will be fully responsible for recruiting and selecting peer role models to participate in the programme. Schools will be advised that the number of peer role models that can be involved is flexible and that this can depend on the level of interest in the opportunity, as well as current COVID-19 restrictions and bubbles. If fewer numbers are recruited all peer role models may wish to attend all sessions but in the event of several peer role models the school may wish to alternate attendance.
6. How	Primary schools will be notified by email upon randomisation to the intervention group and will gain access to the intervention manual along with a programme timetable and list of community contacts via the trial manager. Each school will oversee the timetable planning and subsequent delivery by community role models. Secondary schools will be notified as to whether their primary feeder school has been allocated to receive the intervention or control. In the event of intervention assignment, an introductory email will be sent to both the primary- and secondary lead teachers to formalise and facilitate the link for peer role model provision at each session. The community role model will be provided with the school’s contact details, address, session timings and approximate number of attendees by the study manager.
7. Where	Participants will access the intervention on the primary school premises.
8. When and how much	The programme will consist of a one-hour physical activity after-school session delivered each week for 6 weeks. There will be no cost to participate.
9. Tailoring	All participants will receive the same overarching programme structure; six 1-h sessions delivered over consecutive weeks on the school premises. In each intervention primary school, the local community physical activity provision is mapped to identify potential role models, and through communication with community providers and other physical activity stakeholders a timetable for the intervention is developed. This is done in collaboration with the school so that the interests and choices of the target population are taken into consideration when arranging the timetable.

The intervention logic model (see Additional File 2) displays the intervention planned components and how they link to the intended outcomes. In order to support organisation and delivery of the intervention, intervention manuals and guidelines are provided to the coordinating teacher in each primary and secondary school as well as the community and peer role models (see Additional File 3 for an overview of contents).

### Recruitment and consent

#### School recruitment and retention

State funded schools in South and Mid Wales, UK, will be invited to take part in the study. Six secondary schools will first be invited via the School Health Research Network (SHRN) and recruited to the study. These secondary schools will recruit secondary school students as peer role models to support intervention delivery in primary schools receiving the intervention. Each school will be asked to sign a memorandum of understanding to indicate their agreement to study participation. The memorandum will outline research processes and timelines, as well as roles and responsibilities.

For each secondary school, one feeder primary school will be purposively selected to ensure variation in socioeconomic status (percentage of students eligible for free-school meals), geographic location and school size and using publically available contact details, and invited to take part in the study. In the event that a school declines participation, a subsequent feeder school will be selected and invited to take part. An invitation to participate in the study will be sent to each secondary school’s feeder primary schools via email. A researcher will then contact schools by telephone to discuss the study. Following the same process for secondary schools, selected primary schools will also be asked to sign a memorandum of understanding. To maintain engagement, encourage retention and to thank schools for their time, they will be provided with quarterly newsletters from the time of allocation and a £200 donation following successful completion of the data collection activities. Schools will also receive a quarterly study newsletter, summarising study progress, in order to maintain school engagement, particularly for those schools allocated to control.

#### Child recruitment and parental consent

Two main forms of data collection will be conducted at baseline and again at a 12-month follow-up:

1. A survey of self-reported outcome measures
2. Collection of 7-day accelerometry data (i.e. GT3X ActiGraph accelerometer, which is a non-invasive physical activity monitoring device worn on the waistband).

Recruited primary schools will be asked to invite all girls in year 5 (ages 9–10) to participate in the study at least 2 weeks prior to the data collection date. Year 5 girls will be provided with an information sheet, fully informing them of the study. Simultaneously, all the parents/carers of year 5 girls will be sent an electronic information sheet, fully informing them of the study and giving them the opportunity to opt their child in to the study. The information sheets will detail all possible aspects of their child’s participation in the study, including the completion of a short online survey in school and accelerometer and options of withdrawal up to the point of reporting; stating that children can participate in either the survey or accelerometer or both. The information sheet will highlight that the study is about evaluating an after school programme, but which schools are allocated to receive that or act as controls is down to chance. The information sheet will clearly state that their child can still take part in the after-school programme even if they do not participate in the study.

Parents/carers will be encouraged to discuss the information with their child prior to data collection. Parental written opt-in consent will be required for children to take part in the main trial components (survey and accelerometer). Parents/carers will be asked to return the opt-in form if they are happy for their child to participate in any aspect of the study. If a parent/carer does not return an opt-in form to the school by the 2-week deadline, their child will not enter the study. The class teacher will provide daily oral reminders to the children over this period to ensure that they and their parents read the information sheet and also utilise one other usual mechanism for communication (e.g. texts or emails home).

#### Child assent

All year 5 girls who have been opted in by parents/guardians and are in attendance on the day of data collection will be reminded of the research and their potential role in it. This will involve the teacher/online researcher reading a standardised script to the whole class, outlining all aspects pertaining to voluntary nature of the study, confidentiality and withdrawal. The script will also emphasise that pupils can still take part in the after school programme even if they do not take part in the study data collection. Teachers will be advised to distribute the activity monitors sensitively, being mindful of issues relating to body image and physicality. The research team will also provide the option for a researcher to virtually join (due to COVID-19) their data collection session through the use of an online video call.

To reiterate the study aims and assent process, the first page of the survey (whether electronic or paper-based) will include a simple information and assent section for year 5 girls. This will also outline all aspects pertaining to voluntary nature of the study, confidentiality and withdrawal. Information on how to complete the survey and skipping questions will be included. The child will be required to complete the assent statements for both the survey and activity monitor.

Pupils who have not been opted into the study by their parents, or who choose not to take part, will be provided with alternative activities by their teacher. This will also be the case for any boys within the year 5 classroom in the event that teachers need to keep the whole class together during data collection.

#### Sample size

As this study is focused on feasibility, the purpose is to provide estimates of key parameters for a future full-scale trial, rather than to power the current study to detect statistically significant differences. A total of 90 children within 6 recruited primary schools (allowing for an average of 15 girls in year 5) will allow an estimation of feasibility criteria with reasonable precision across a diverse range of context (e.g. intervention fidelity, completion of assessments, and valid primary outcome data). For girls within the intervention schools only (*n*=60), we will estimate that 50% will attend the intervention (i.e. minimum of one session) within ±12.7 percentage points using a 95% confidence interval. Similarly, for girls from all schools (*n*=90), the parameter of 70% returning valid primary outcome data can be estimated within ±9.5%, and 80% will complete baseline/follow-up to within ±8.3%.

#### Randomisation

Randomisation will be completed at primary school-level and will be randomised to the intervention or control group with a fixed ratio of 2:1 schools, respectively. We will stratify by Local Authority (LA), depending on how primary feeder schools are recruited with each LA. If within one LA not enough schools are recruited to achieve a 2:1 ratio, we will ensure balance by reverting to a 1:1 ratio ensuring that at least one schools is allocated to each arm within an LA. An independent statistician at Cardiff University Centre for Trials Research (CTR) will randomise the schools after all six schools are recruited to the study to minimise drop out after randomisation. If a school withdraws before baseline data collection, they will be replaced with another randomly selected school from the same strata and retain the allocation of the school that withdrew. If a school withdraws after baseline data collection and the school has been informed of intervention allocation but none of the intervention has been delivered, the school’s baseline data collection will be reported and included in the ITT analysis. If a school withdraws after baseline data collection and they have started the intervention, then they are not replaced. The school will be followed-up as normal unless the school withdraws fully from the trial and follow-up at 12 months. Allocations will be blinded from the trial statistician and health economist. The schools will be informed of the allocation after baseline data is collected from the students. All study staff and participants will be blinded at baseline data collection.

#### Outcome measures

The outcomes that will be reported in this feasibility study are as follows:

##### Primary outcomes

- Average daily minutes spent in moderate-to-vigorous physical activity (MVPA) measured using 7-day accelerometer data.

##### Secondary outcomes

- Number of schools and children recruited, randomly allocated and retained at 12-month follow-up measured using recruitment data, attendance registers and interviews.
- Assessment of the feasibility and acceptability of the evaluation design and methods for a future full-scale trial measured using interviews and focus groups.
- Assessment of intervention fidelity (delivery) measured using teacher logbooks, attendance registers and session observations.
- Assessment of intervention acceptability to children (girls and boys), parents, school staff and role models measured through interviews and focus groups
- Assessment of the feasibility of conducting an economic evaluation in a future full-scale trial using a pilot cost consequence analysis, testing pupil self-reported outcomes measures (Child Health Utility-9D (CHU-9D) [21] and EQ-5D-Y [22]).
- Assessment of sedentary time and time spent at different physical activity intensities within specific segments (e.g. during the club, after school or at weekends) measured using 7-day accelerometer data at baseline and 12-month follow-up.

### Progression criteria for a potential future trial

Criteria for progression to a full-scale trial will be used to inform a decision on whether and how to proceed. Each progression criterion (PC) is outlined below.

1. The intervention is implemented with fidelity (in a manner in line with intervention theory) in at least 3 of 4 intervention schools.
2. At least 50% of assenting children in the intervention schools attend 50% of the scheduled sessions.
3. The process evaluation indicates the intervention is acceptable to children, parents, school staff and role models.

Progression criteria relating to obtaining data regarding completion of outcome measures will be assessed using the following progression criteria:

4. At least 3 of 4 intervention schools and 1 of 2 control schools are retained throughout the study. At least 80% of children approached complete the baseline and follow-up accelerometer measures. Proceed: 80% of children complete baseline and follow-up accelerometer measure; Stop: < 50%; Review: 50–79% of children complete.
5. At least 70% of recruited children who receive an accelerometer return valid data (>3 days of 600 minutes including 1 weekend day) at baseline and follow-up for the primary outcome measure. Proceed: 70% or more; Stop: <40%; Review: 40–69%

These criteria have been agreed in advance of data collection with the Trial Steering Committee (TSC). The TSC will consider the quantitative and qualitative data [23] to review and make an overall judgement on whether the intervention can be delivered with fidelity. Potential impacts of COVID-19 will be considered as well as risk of generalisability bias [24]. In line with development guidance for feasibility studies [25], the study does not aim to provide an indication of the effectiveness of the intervention; given the small, purposively selected sample, such an estimate would not be meaningful.

### Data collection

In the current climate, many (if not all) schools will not allow non-staff members access to their premises. Therefore, minimal face-to-face data collection methods have been included within the current project. Data collections will be mainly teacher-led (with virtual attendance by an online researcher where requested) and an electronic survey method offered. Where data collection materials need to be provided to the school (e.g. activity monitors and paper surveys), we have developed a safe contactless delivery approach.

Data collection will be overseen by the class teacher in normal class time. Teachers will be provided with a study introduction sheet which contains a script that they will read out to their pupils, along with some ‘dos’ and ‘don’ts’.

### Classroom report

Using a standardised template, the teacher will be asked to provide count information on the number of parental opt-ins, number of absentees and the number of pupil opt-outs (via hard copy or electronic).

### Survey

The survey will be made available in English and Welsh and will be self-completed by pupils within the classroom under teacher supervision during school hours. Data collection will be facilitated by electronic data capture using a bespoke Qualtrics database, and paper copies of the survey will also be made available. Schools will be asked when signing up to the study if they would prefer electronic or pen and paper format. Each child opted into the study by their parents/carers will be allocated a unique Participant ID ahead of data collection, ensuring all participant data are pseudonymised.

The survey will collect data on demographics (age, gender and ethnicity), self-reported physical activity (Physical Activity Questionnaire for Older Children (PAQ-C scale)) [26], psychosocial outcomes (activity-based perceptions of autonomy, relatedness, competence and enjoyment), current after-school sport or physical activity club attendance (school and community) and health-related quality of life ((CHU-9D [21] and the EQ-5D-Y) [22]). Three schools will complete the EQ-5D-Y, and three schools the CHU-9D to reduce burden on pupils completing the questionnaires, whilst retaining the ability to answer feasibility questions. All questions will be closed response, with an ‘I do not want to answer’ option provided throughout. For electronic survey collections, the software will enable partial survey completions to be captured.

### Activity monitor

Participants will also be asked to wear a GT3X ActiGraph accelerometer on the right hip for seven consecutive days (during waking hours) and complete a monitor wear diary. Activity monitors will be clearly numbered, charged and initialised for assessment. Each child opted in by their parents/carers will receive an activity monitor, an activity monitor diary and parental instructions. The activity monitor diary will ask participants to keep a record of the time they removed the belt, reason for removal and time they placed the belt back on. This will allow any water-based activities such as swimming to be captured. Participants will return their monitor and diary to school after 7 days following which study items will be collected by the research team before the data is downloaded and securely saved for processing.

### Follow-up

Twelve months after baseline data collection, both sets of data collection modes outlined above will be repeated as a follow-up assessment. Three weeks prior to this, schools will send a reminder to parents/guardians to make them aware of the remaining data collection in school. Processes described above for electronic survey and accelerometry data collection will be repeated in the following week.

### Withdrawal and loss to follow-up

Participants will be able to withdraw from the study up until publication of the results. Our default position if a participant withdraws from the study but does not request destruction of their data will be to retain data already collected; however, the participant will have the option to withdraw their data as outlined in the participant information sheets. The main trial information sheets will advise pupils, parents and carers on withdrawal procedures in relation to both the survey and accelerometer data; namely that the survey records data entered throughout and therefore pupils will need to specify to their teacher or parents/carers that they wish to withdraw their survey data, who will then inform the research team. Pupils will be advised to also tell their teacher or parents/carers if they wish to withdraw their activity monitor data, so that they can again inform the research team to organise withdrawal. Once a participant has withdrawn no further data will be collected from them.

### Process evaluation

In accordance with the Medical Research Council framework [27], the embedded process evaluation will explore intervention acceptability and the feasibility of implementing the intervention (including the extent to which the intervention was delivered as planned) and aim to understand mechanisms from the perspectives of participants and others and explore the context in which the intervention was conducted. Exploration of context will include the impact of the COVID-19 pandemic to separate out what is happening due to the context of COVID and what might be transferable to non-COVID times. The process evaluation will use both quantitative and qualitative measures as outlined below.

### Quantitative methods

At each intervention school the teacher overseeing intervention delivery will complete a weekly attendance register to record pupil attendance across sessions. The teacher will also complete a delivery logbook, using a standardised template, to assess whether community role models delivered the planned components (see Additional File 2) of each session fully, partially or not at all (i.e. fidelity scores). The focus on delivery of the components will be with regards the function of the components in line with the intervention theory, rather than specific form of delivery. A project team member will observe two randomly selected sessions in each school also completing the logbook template as a form of verification of teacher-assessed fidelity scores.

### Qualitative methods

At each intervention school, one-to-one interviews with teachers overseeing intervention delivery (1 per school), head teachers or member of the senior leadership team (1 per school), parents of participating girls (2 per school) and community role models (4 per school) will be conducted. Interviews will explore acceptability and feasibility of implementation of the intervention.

Three focus groups (involving 6 pupils in each) will be conducted at each intervention school with a sample of girls who have participated in the intervention and a separate group of year 5 boys. The focus group will be conducted at two-time points with the sample of girls, immediately post-intervention and 3 months later to explore experiences of the intervention, acceptability and barriers and facilitators to participation and longer term experiences of physical activity beyond intervention receipt. The focus group with boys aims to explore acceptability and potential unintended consequences of a targeted intervention for girls.

All peer role models will be invited to participate in a focus group at the end of intervention delivery to explore feasibility and acceptability of the peer role model component of the intervention and barriers and facilitators to involvement. We will also explore perceptions of ‘how’ the intervention is perceived to have worked or not and gather contextual considerations (e.g. COVID context and what’s specific to that, transferable beyond it).

### Adverse events

A risk assessment has found this trial to be low risk. All schools will be asked to notify the research team of any serious instance which is perceived to be study-related by submitting an anonymised version incidence report form. Any safeguarding issues arising during data collection will be reported immediately (and within 24 h of knowledge of the event) to the main school contact. The PI will continue to monitor reports and determine whether they are adverse events (AEs) or serious adverse events (SAEs). There are no expected adverse events related to the CHARMING Programme or research procedures.

### Data analysis

#### Statistical analysis

The main outcomes in this feasibility trial are the rates of consent and recruitment (schools, peer-role models, year 5 girls), and retention at 12-month follow-up. Analysis of these data will be mainly descriptive (means and SD, median and 25th to 75th centiles for non-normal distributions, or *N* and %) as appropriate. Descriptive comparisons of these data will be made between intervention and control arms. Loss to follow-up in intervention and control groups will be reported. Demographic characteristics will be summarised descriptively as appropriate. We will characterise individuals that are lost to follow up at 12 months (with respect to demographics and intervention engagement.

For schools receiving the intervention, summary statistics will be presented for intervention reach and fidelity measures overall and by school/LA. This will include the proportion of recruited girls attending the first session, a minimum of one session and the number of session attendances per individual (max of 6). Mean (SD) or median (25th to 75th centiles) attendance per session will be calculated and summary statistics will be calculated on peer-role model engagement (e.g. attendance rates). Session attendance and presence or absence of the intervention components (role model delivering and participating in each session, question and answer opportunity with role models post-session, sign-posting) will be presented over time, overall and by school.

Accelerometry data will be analysed using a batch processing protocol; continuous periods of 60 min of zero counts will be considered as ‘non-wear time’ and removed. All data between 23:00 and 06:00 will be removed to ensure focus on awake time. Participants will be included in the analysis if they provide a minimum inclusion of 3 valid days. The percentage of original participants retained to 12-month follow-up who provide valid useable accelerometry data will be reported. Average counts per minute (CPM) will be used as a measure of total physical activity. Using the Evenson cut-points [28], the average daily minutes spent sedentary (cut-point of < 100 CPM), in MVPA (≥ 2296 CPM) and time-segment-specific time spent in each activity intensity (e.g. during club, after school or at weekends will be estimated using 7-day accelerometer data and summarised overall and by trial arm. Mixed-effects linear regression will be used to estimate direction of intervention effects on accelerometer-measured physical activity on the adjusted mean difference between intervention and control groups, alongside a 95% confidence interval (CI). School-level variance will be accounted for by inclusion of school as a random effect, and any remaining differences in the baseline assessment of the outcome measure, and local authority area (by which the randomisation was stratified), as fixed factors. The school-level intra-class correlation (ICC) coefficient for average daily minutes of MVPA over the 7 days will be estimated alongside a 95% CI. Whilst likely non-significant due to limited power, this should be in the direction of a favourable intervention effect.

Response rates and level of completion of the survey measures such as (self-reported physical activity (PAQ-C scale), psychosocial outcomes (activity-based perceptions of autonomy, relatedness, competence and enjoyment) and current after-school sport or physical activity club attendance (school and community), at baseline and 12-month follow-up will be reported using descriptive statistics. Regression models will also be used to estimate direction of intervention effects on 12-month survey outcomes. Mixed-effect models will take the clustering of individuals in schools into account using a random model and adjust for baseline outcomes.

All analyses will be intention to treat (i.e. students will be analysed in the groups in which their school was randomised to, regardless of adherence to the intervention) and missing outcome data will not be replaced. The analysis and reporting of this cluster randomised controlled trial will be in accordance with CONSORT (Consolidated Standards of Reporting Trials) guidelines {Eldridge, 2016 #354}.

#### Economic analysis

A Health Economics Analysis Plan (HEAP) in accordance with the main statistical analysis plan will set out the objectives and methods for data collection and analysis of the health economics findings. A pilot cost consequence analysis will test the feasibility of conducting an economic evaluation in a future full-scale trial. Response rates and level of completion of the economic measures of EQ-5D-Y, CHU-9D at baseline and 12-month follow-up will be reported using descriptive statistics taking account of clustering. Ceiling effects in the health-related quality of life measures EQ-5D-Y and CHU-9D will be assessed.

During teacher interviews at intervention schools, questions will be added to explore if it is feasible to identify and measure the resources required to deliver the intervention to estimate a cost per programme and per participant, using micro-costing methodology used successfully in previous studies [29, 30].

Topic Guide questions will inquire about time taken to set up and delivery the intervention, such as time to recruit role models, session planning, and liaising with local organisations to deliver sessions. We will liaise with teachers responsible for intervention delivery to gather this information, including information on teacher salary band.

Teacher costs will be sourced from the National Union of Teachers (NUT) [31] pay structure for qualified classroom teachers in Wales. School based costs will be collected from a Local Education Authority perspective, accounting for on-costs (e.g. national insurance and pension costs), and using the cost year 2020–2021.

Parents taking part in the one-to-one interviews will be asked about their willingness to answer questions regarding their child’s use of primary care such as the GP, nurse, and hospital services, and whether there are other services they feel are important to include. Whether parents would be willing to complete a questionnaire at study time points (e.g. at baseline and follow up) and what format they would prefer the questionnaire to be in (e.g. paper or online). These questions would help inform the development of a Client Service Receipt Inventory (CSRI) as part of a cost-effectiveness analysis in a subsequent definitive randomised controlled trial.

All analyses will be conducted using intention to treat analysis (i.e. students will be analysed in the groups in which their school was randomised to, regardless of adherence to the intervention) and missing outcome data will not be replaced. The health economics component of the study will be written up in accordance with the Consolidated Health Economic Evaluation Reporting Standards (CHEERS) statement [32].

#### Qualitative analysis plan

Interviews and focus groups will be recorded using an encrypted audio recorder and stored on password-protected computers. All recordings will be transcribed verbatim and fully anonymised prior to analysis. Computer software NVivo v12 will be used to manage and analyse qualitative data and transcripts. Thematic analysis will be used to analyse all focus groups and interviews. Thematic analysis of focus groups and interviews will examine acceptability, feasibility, fidelity and engagement. Triangulation of the process evaluation data will be used to combine qualitative and quantitative data analysis. For questions specific to the health economic analysis, the anonymised transcribed sections of these responses will be sent securely to the health economist, who will manage the analysis of the responses to explore the acceptability and feasibility of a CSRI and intervention costing survey. If there is consensus in the data as to what questions/items that should be included in these questionnaires, this will be reported to help inform a future cost-effectiveness analysis.

#### Trial management

The sponsor is Cardiff University. The study will be supported by the Cardiff University CTR, which is a fully registered Clinical Trials Unit. The Sponsor had no role in the design of this trial and will not have any role during its execution, analyses, data interpretation or decision to submit results.

#### Study management committee

The Study Management Group (SMG) will meet monthly throughout the course of the study and will include the chief investigators, co-applicants, collaborators and study manager. The SMG will be responsible for overall trial management and ensuring the study adheres to the protocol.

#### Trial steering committee

A TSC will meet approximately every quarter to provide study oversight. The TSC comprises eight members in total. These include two independent academics (one of whom is the Chair), an independent statistician, two policy leads, a deputy head teacher and the study principal investigator (PI) and study co-PI. The TSC will provide independent oversight for the study.

### Data management

#### Quality control

Data from the paper surveys will be entered into the Qualtrics database by the Trial Manager (TM) and/or Data Manager (DM) retrospectively. Quality checks will take place every 5 participants entries, 50% of which will be quality checked and for which error rates will be calculated. The acceptable rate of error will be <1% and should the calculated percentage exceed this, and the trial team will be trained again.

#### Data protection and participant confidentiality

Access to the Qualtrics database will only be given to the TM or DM. Should paper surveys be used, these will be held separately and securely in a locked cupboard, with access limited to essential research team members. All study data will be anonymised, stored securely and password protected. All data will be securely stored on Cardiff University’s internal server with secure transfer between team members. All procedures for data collection, handling, storage and management will comply with GDPR. Anonymised quotations will be used in reporting results. Only the trial team will have access to the final dataset. All research staff involved will have up to date GCP training.

#### Auditing

No independent audits are planned.

#### Confidentiality

All data will be kept for 15 years in line with Cardiff University’s Research Governance Framework Regulations for clinical research. Electronic data will be stored confidentially on password-protected servers maintained on University networks. All hard copy forms will be stored in locked filing cabinets. Audio files for qualitative interviews and focus groups will be recorded on encrypted audio-recorders and securely held in password-protected servers maintained on university networks. They will be transcribed and pseudonymised using university-approved transcription companies. No identifiable data will be published.

#### Dissemination policy

A publication plan and dissemination policy will be written. Study results will be disseminated through peer-reviewed scientific journals. Two publications are expected, one reporting quantitative and health economic outcomes and the second reporting process evaluation outcomes. On completion of the study a final report will be prepared for Health and Care Research Wales. The study results will be disseminated in full and with a lay summary on the DECIPHer websites, and a summary of the results will be disseminated to all schools. Any data requests should be made to DECIPHer.

#### Public involvement

During the development of this study, we consulted with a young people’s advisory group (ALPHA) to inform the refinement of the intervention and identification of peer role models. The Youth Sports Trust Young Ambassadors are providing ongoing insights into the recruitment of peer role models and community provision through a series of consolation events. A primary school has been identified as a Public Involvement school to inform the development of process evaluation tools.

## Discussion

Despite the need for a physical activity intervention targeting girls within the primary school setting there is currently a significant gap in the evidence base and availability of suitable programmes. The CHARMING Study directly addresses this gap. The results of this feasibility study will contribute to the evidence base on improving outcomes for young girls physically activity, including potential unintended consequences of targeting intervention at girls only. In addition, the findings from this feasibility study will determine the progression to a full-scale trial to assess the effectiveness and cost-effectiveness of the CHARMING Programme and will inform parameters of study design specifically recruitment processes, methods of data collection and choice of outcome measures.

### Study status

Current protocol: FINAL. Recruitment start date: 01 03 21. Approximate recruitment end date: 01 10 21.

## **Supplementary Information**

**Additional file 1.** SPIRIT Checklist.

**Additional file 2.** Intervention logic model.

**Additional file 3.** Overview of contents contained within Study Manuals and Guides.

## Data Availability

Not applicable (Protocol Paper).

## Abbreviations

AEs: Adverse events
CHEERS: Consolidated Health Economic Evaluation Reporting Standards
CHU-9D: Child Health Utility-9D
CI: Confidence interval
CONSORT: Consolidated Standards of Reporting Trials
CSRI: Client Service Receipt Inventory
CTR: Centre for Trials Research
DM: Data manager
HEAP: Health Economics Analysis Plan
ICC: Intra-class correlation
LA: Local authority
MVPA: Moderate to Vigorous Physical Activity
NICE: National Institute for Health and Care Excellence
NUT: National Union of Teachers
PAQ-C: Physical Activity Questionnaire for Older Children
PC: Progression criteria
PI: Principal investigator
SAEs: Serious adverse events
SD: Standard deviation
SDT: Self-determination theory
SHRN: School Health Research Network
SMG: Study management
SPIRIT: Standard Protocol Items: Recommendations for Interventional Trials
TM: Trial manager
TSC: Trial Steering Committee
WHO: World Health Organization
