# Supplementary material for: CHoosing Active Role Models to INspire Girls (CHARMING): protocol for a cluster randomised feasibility trial of a school-based, community-linked programme to increase physical activity levels in 9–10-year-old girls
Source: Pilot Feasibility Stud. 2022 Jan 3;8:2. doi: 10.1186/s40814-021-00961-6 (PMC8720937; doi:10.1186/s40814-021-00961-6)
Supplement: Supplementary file 1 — Additional file 1. SPIRIT Checklist. [file 40814_2021_961_MOESM1_ESM.docx]

**Additional File 1 – Study Manuals and Guides: Content overview**

1. **CHARMING STUDY MANUAL – SECONDARY SCHOOLS**
2. Advertising the opportunity

## Roles and Responsibilities of Peer Role Models

1. Teachers responsible for recruitment
2. Gaining consent from parents
3. Attending the CHARMING programme
4. Focus groups with Peer Role Models
5. **CHARMING STUDY MANUAL – PRIMARY SCHOOL DATA COLLECTION**
6. Data collection timetable
7. Sending out the Parental Opt-in forms and Child Information Sheets
8. Provide Information to the Research Team
9. Preparing for Data Collection
10. Study Boxes
11. On the Day of Data Collection
12. Collection of the Activity Monitors
13. Withdrawal
14. Randomisation
15. Follow Up Data Collection
16. **CHARMING STUDY MANUAL – INTERVENTION PRIMARY SCHOOLS**
17. Programme timetable and community contacts for scheduling provision
18. Data Collection during CHARMING Programme Delivery
19. Safeguarding
20. Focus Groups
21. Interviews
22. **CHARMING PEER ROLE MODELS – ROLES AND RESPONSIBILITIES**
23. Overview of the programme
24. What experience do you need?
25. What will you get from being a Peer Role Model for the CHARMING Programme?
26. Roles and responsibilities
27. Code of conduct
28. When the CHARMING programme finishes
29. **COMMUNITY ROLE MODELS – ROLES AND RESPONSIBILITIES**
30. Overview of the programme
31. What will your role involve?
32. Code of conduct
33. When the CHARMING programme finishes
