# Supplementary material for: CHoosing Active Role Models to INspire Girls (CHARMING): protocol for a cluster randomised feasibility trial of a school-based, community-linked programme to increase physical activity levels in 9–10-year-old girls
Source: Pilot Feasibility Stud. 2022 Jan 3;8:2. doi: 10.1186/s40814-021-00961-6 (PMC8720937; doi:10.1186/s40814-021-00961-6)
Supplement: Supplementary file 2 — Additional file 2. Intervention logic model. [file 40814_2021_961_MOESM2_ESM.docx]

**Additional File 2 – CHARMING Intervention logic model**

- Peer role models from adjoining secondary school
- 1-hour weekly physical activity taster sessions (after-school for 1 term)

- Community role model delivering the session

- Peer role model participating in sessions
- Post-session Q&A session with role model
- Signposting to community activities/clubs

**Intermediate**

- Increased enjoyment of school physical activity
- Improved self-efficacy, attitudes, skills and knowledge relating to physical activity
- Facilitate school-transition

**Cognitive processes**

- Awareness of community physical activity opportunities
- Developed an interest in being more physically active

**Behavioural processes**

- Increase socialisation with peers and role models
- Increased confidence and self-efficacy
- Experience of new physical activity/sport
- Intention to take up new activity/join club
- Increased physical activity levels

SHORT-TERM OUTCOMES

OUTCOMES

- School-community partnerships
- Mapping of community physical activity provision
- Transport options

PLANNED COMPONENTS

INPUTS

- Community role models
- Appealing activities
- Equipment and space on school premises

**Long-term**

- Frequent attendance at a community club/activity
- Sustained physical activity
- Improved children’s fitness
- Reduced sedentary time

**Displacement**

- Does intervention attendance replace other physical activity?
